# Unravelling the potential mechanisms behind hospitalization-associated disability in older patients; the Hospital-Associated Disability and impact on daily Life (Hospital-ADL) cohort study protocol

**DOI:** 10.1186/s12877-016-0232-3

**Published:** 2016-03-05

**Authors:** Lucienne A. Reichardt, Jesse J. Aarden, Rosanne van Seben, Marike van der Schaaf, Raoul H. H. Engelbert, Jos A. Bosch, Bianca M. Buurman, on behalf of the Hospital-ADL study group

**Affiliations:** Department of Internal Medicine, Section of Geriatric Medicine, Academic Medical Center, PO Box 22600, 1100 DD Amsterdam, The Netherlands; Department of Rehabilitation, Academic Medical Center, Amsterdam, The Netherlands; Amsterdam Center for Innovative Health Practice (ACHIEVE), Faculty of Health, Amsterdam University of Applied Sciences, Amsterdam, The Netherlands; Department of Clinical Psychology, University of Amsterdam, Amsterdam, The Netherlands

**Keywords:** Acute hospitalization, Hospitalization-associated disability, Functional decline, Older patients

## Abstract

**Background:**

Over 30 % of older patients experience hospitalization-associated disability (HAD) (i.e., loss of independence in Activities of Daily Living (ADLs)) after an acute hospitalization. Despite its high prevalence, the mechanisms that underlie HAD remain elusive. This paper describes the protocol for the Hospital-Associated Disability and impact on daily Life (Hospital-ADL) study, which aims to unravel the potential mechanisms behind HAD from admission to three months post-discharge.

**Methods/design:**

The Hospital-ADL study is a multicenter, observational, prospective cohort study aiming to recruit 400 patients aged ≥70 years that are acutely hospitalized at departments of Internal Medicine, Cardiology or Geriatrics, involving six hospitals in the Netherlands. Eligible are patients hospitalized for at least 48 h, without major cognitive impairment (Mini Mental State Examination score ≥15), who have a life expectancy of more than three months, and without disablement in all six ADLs. The study will assess possible cognitive, behavioral, psychosocial, physical, and biological factors of HAD. Data will be collected through: 1] medical and demographical data; 2] personal interviews, which includes assessment of cognitive impairment, behavioral and psychosocial functioning, physical functioning, and health care utilization; 3] physical performance tests, which includes gait speed, hand grip strength, balance, bioelectrical impedance analysis (BIA), and an activity tracker (Fitbit Flex), and; 4] analyses of blood samples to assess inflammatory and metabolic markers. The primary endpoint is additional disabilities in ADLs three months post-hospital discharge compared to ADL function two weeks prior to hospital admission. Secondary outcomes are health care utilization, health-related quality of life (HRQoL), physical performance tests, and mortality. There will be at least five data collection points; within 48 h after admission (H_1_), at discharge (H_3_), and at one (P_1_; home visit), two (P_2_; by telephone) and three months (P_3_; home visit) post-discharge. If the patient is admitted for more than five days, additional measurements will be planned during hospitalization on Monday, Wednesday, and Friday (H_2_).

**Discussion:**

The Hospital-ADL study will provide information on cognitive, behavioral, psychosocial, physical, and biological factors associated with HAD and will be collected during and following hospitalization. These data may inform new interventions to prevent or restore hospitalization-associated disability.

## Background

Studies have observed that at least 30 % of older patients hospitalized with an acute medical illness show a persistent decline in their ability to maintain Activities of Daily Living (ADLs) [1–5]. Such activities are prerequisites to self-care and independent living and include bathing, dressing, transferring out of bed, eating, toileting, and being mobile in and around the house. [1, 3–7]. This decline has been denoted hospitalization-associated disability (HAD), and is defined as the loss of ability to perform one or more of the basic ADLs [6].

HAD is an important problem; it is the leading cause of loss of independence at older age [4] and it is a complex and highly dynamic process with possible recurrent disability episodes in older patients [6, 8]. Research shows that older persons who have been hospitalized have a 60-fold increased risk to develop permanent disabilities [9]. The first month after hospital discharge has been identified as a critical period for recovery, after which disabilities have a high risk of becoming permanent [3]. Moreover, patients with new disabilities are at high risk for other adverse outcomes within three months post discharge: 20 % have readmissions [10], and post-discharge mortality is 25 % [1, 3, 11, 12]. In light of the high prevalence in older patients, and the rapid aging of western societies with a concomitant rise in hospitalizations, a better understanding of HAD is urgently needed.

Previous research has been able to identify a number of generic risk factors for hospitalization-associated disability such as older age [5], the severity of acute illness, geriatric conditions, cognitive impairment and delirium [1, 6, 13, 14]. However, a more fine-grained analyses and characterization of potentially modifiable risk factors is virtually absent from the literature. Little is known, for example, about: 1] the course of loss of muscle mass and strength, and the amount of physical activity older patients perform; 2] the association of cognitive, (psycho-)somatic, behavioral, and psychological restrictive symptoms with the onset and recovery from HAD within the critical period of three months post-discharge such as cognitive impairment, fatigue, pain, sleep quality, shortness of breath, dizziness, fear of falling, diminished self-efficacy, apathy, depression, and anxiety and; 3] the association of the inflammatory activity and related sickness behaviors with the onset and recovery from HAD. Moreover, most previous studies have utilized relatively long follow-up intervals (e.g., from admission to three months or more) [1–3, 15]. Thus information is lacking on events and processes that take place during the weeks after discharge, which are thought to be critical to recovery.

The current study – Hospital-Associated Disability and impact on daily Life (Hospital-ADL study) – aims to investigate cognitive, behavioral, psychosocial, physical, and biological factors that may be associated with HAD in acutely hospitalized older adults, performing frequent assessments to capture their dynamic development from hospital admission to three months post-discharge. This overall aim can be separated into the following five subordinate aims:

1. To study the temporal profile of HAD (i.e., loss of ADL) from hospitalization to three months post-discharge.
2. To investigate the course of physical functions that are essential to support ADL, such as muscle mass, muscle strength, and physical performance and spontaneous activity, and test its predictive value for the onset and recovery from HAD, health care utilization, and health-related quality of life (HRQoL) at three months post-discharge.
3. To study the prevalence, incidence and course of cognitive, (psycho-)somatic, behavioral, and psychological problems older patients experience from hospitalization up to three months post-discharge that might be restrictive in recovery from HAD post-discharge such as cognitive impairment, fatigue, pain, sleep quality, shortness of breath, dizziness, fear of falling, diminished self-efficacy, apathy, depression, and anxiety.
4. To study the association of aforementioned symptoms with HAD, health care utilization, and HRQoL.
5. To study the association of metabolic and proinflammatory factors, and physical and behavioral concomitants (e.g., sickness behaviors, loss of muscle mass) with the onset of and recovery from HAD, health care utilization, and HRQoL.

## Methods/Design

### Study design and setting

The Hospital-ADL study is a multicenter, observational, prospective cohort study designed by an interdisciplinary team of researchers in the field of geriatrics, nursing, psychology, physical therapy and rehabilitation. Six hospitals will participate: 1] the Academic Medical Center in Amsterdam (AMC), a 1002-bed university teaching hospital; 2] the Isala in Zwolle, a 994-bed regional teaching hospital; 3] the Tergooi in Blaricum, a regional teaching hospital (633-bed spread over two sites: Hilversum and Blaricum); 4] the Slotervaart Hospital in Amsterdam, a 310-bed regional teaching hospital; 5] the BovenIJ Hospital in Amsterdam, a 313-bed regional teaching hospital, and; 6] the Meander Medical Center in Amersfoort, a 543-bed regional teaching hospital. The study has started October 1, 2015 and will end after the last patient has been followed up for three months post-discharge. We expect the recruitment phase to be completed late 2016.

### Patients

We aim to recruit 400 non-fully disabled adults aged ≥70 years. The following inclusion criteria apply: 1] acutely admitted at departments of Internal Medicine, Cardiology or Geriatrics for 48 h or more in one of the above mentioned hospitals; 2] 70 years and older; 3] have approval from the attending Medical Doctor for inclusion; 4] score of 15 or higher on the Mini-Mental State Examination; 5] Dutch language proficiency sufficient to complete questionnaires. Patients will be excluded if they: 1] have a life expectancy of three months or less as assessed by the attending Medical Doctor, or; 2] are disabled in all six basic ADL as determined by the Katz-ADL index [16].

### Procedures

Eligible patients will be contacted, and the patient will be informed about the objectives of this study and the study procedures, upon which written informed consent is obtained. Furthermore, a legal representative of the patient will be contacted if the patient has a MMSE score between 15 and 20. Two mobile geriatric assessment teams will visit all six hospitals and will be present on Monday, Wednesday and Friday for consenting and to perform assessments. The mobile geriatric assessment teams consist of a psychologist, physical therapist, and/or a health scientist. The teams are trained in the study procedures of obtaining informed consent, to perform assessments and physical performance tests with adequate inter- and intra-rater reliability (>0.8), and completing the electronic case report form (eCRF).

Table 1 provides an overview of the location, content of assessment and duration of data collection per time point. There will be at least five data collection points; within 48 h after admission (H_1_), at discharge (H_3_), and at one (P_1_; home visit), two (P_2;_ by telephone) and three months (P_3;_ home visit) post-discharge. If the patient is admitted for more than five days, additional measurements will be planned during hospitalization on Monday, Wednesday, and Friday (i.e., the days that the mobile geriatric assessment team is present) (H_2_),

**Table 1:** Time, location, content of assessment and duration of the Hospital-ADL study

Time	Location	Content of assessment	Duration (minutes)
H_1_ (Within 48 h after admission)	Hospital	Medical & demographical data	60
		Socio-demographic characteristics	
		Medical comorbidity	
		Geriatric conditions	
		Severity of acute illness (medical record)	
		Personal interview/self-report data	
		Cognitive functioning	
		ADL/physical functioning	
		Behavioral & psychosocial functioning	
		Health care utilization (medical record)	
		Physical performance tests	
		Blood parameters	
H_2_ (During hospital stay on Monday-Wednesday-Friday)	Hospital	Medical & demographical data	20–30
		Severity of acute illness (medical record)	
		Short personal interview/self-report data	
		Physical performance tests	
		Blood parameters	
H_3_ (At hospital discharge)	Hospital	Personal interview/self-report data	40
		Cognitive functioning	
		ADL/Physical functioning	
		Behavioral and psychosocial functioning	
		Physical performance tests	
		Blood parameters	
P_1_ (One month post-discharge)	Home visit	Medical & demographical data	60
		Socio-demographic characteristics	
		Geriatric conditions	
		Personal interview/self-report data	
		Cognitive functioning	
		ADL/physical functioning	
		Behavioral & psychosocial functioning	
		Health care utilization	
		Physical performance tests	
P_2_ (Two months post-discharge)	By telephone	Personal interview/self-report data	20
		ADL/physical functioning	
		Behavioral and psychosocial functioning	
		Health care utilization	
P_3_ (Three months post-discharge)	Home visit	Medical & demographical data	60
		Socio-demographic characteristics	
		Geriatric conditions	
		Personal interview/self-report data	
		Cognitive functioning	
		ADL/physical functioning	
		Behavioral & psychosocial functioning	
		Health care utilization	
		Physical performance tests	
		Mortality (medical record)	

Data will be collected through: 1] medical and demographical data (e.g., socio-demographic characteristics, severity of acute illness, and geriatric-, and chronic conditions); 2] personal interviews (including cognitive, behavioral, psychosocial, and physical parameters, and health care utilization, see description of information collected below); 3] physical performance tests (e.g., gait speed, muscle strength, muscle mass, mobility and physical functioning, see below) and; 4] blood samples (e.g., to assess markers of inflammation).

The personal interviews will take place during hospitalization (H_1_, H_2_, and H_3_), at the participant’s home or residence (P_1_ and P_3_; one and three months post-discharge), and by telephone (P_2_; two months post-discharge). Physical performance data will be collected within 48 h after admission (H_1_), during hospitalization on Monday, Wednesday and Friday (H_2_), at discharge (H_3_), and at one and three months post-discharge (P_1_ and P_3_).

#### Primary outcome

The primary outcome is the level of ADL functioning three months post-discharge compared to premorbid functioning, which are measured with the 6-item Katz-ADL index score of the modified Katz-ADL index [17]. The Katz-ADL index score assesses the degree of independence in bathing, dressing, toileting, use of incontinence materials, transfer from bed-chair and eating [16].

#### Secondary outcomes

Secondary outcomes include:

1. Health care utilization (extension of the Minimal Dataset (MDS) [18] and Comprehensive Geriatric Assessment of the Transitional Care Bridge (TCB) [19], see below).
2. Quality of life as measured with the EuroQol-5D [20] and the three items of the MDS [18] (see description below).
3. Physical performance tests (see below for description of included tests).
4. Mortality.

### Scales and assessments

Table 2 gives a detailed overview of the primary and secondary outcomes at each time point.

**Table 2:** Summary of outcome measures and time points of assessment in Hospital-ADL study

	Question or instrument	H_1_	H_2_	H_3_	P_1_	P_2_	P_3_
1. Medical & demographical data							
Age	Date of birth	×*					
Gender		×					
Postal code		×					
Date and time of admission		×*					
Education	(In accordance with Verhage, 1966 [57])	×					
Ethnicity	Country of birth patient and parents	×					
Marital status [18]		×					
Living arrangement [18, 19]		×			×		×
Medical comorbidity	CCI [21]	×*					
Severity of acute illness	MEWS [22]	×*	×*	×*			
Admission diagnosis		×*					
2. Personal interviews/self-report data							
2.1 Cognitive functioning							
Cognitive impairment	MMSE [23]	×		×	×		×
Delirium	CAM [24, 58]	×					
	Assessing whether: 1] the patient needs help with self-care; 2] the patient has previously undergone a delirium and; 3] the patient has a cognitive impairment [25]	×*					
2.2 Behavioral & psychosocial functioning							
Fear of falling	NRS fear of falling	×	×	×	×	×	×
Anxiety	STAI-6 [31]	×		×	×	×	×
Apathy	GDS-15 [29]	×		×	×	×	×
General self-efficacy	ALCOS-12 [34]			×	×	×	
Quality of life	1] In general, how is your quality of life?; 2] How would you grade your life at this moment, with a range between 0 and 10? and; 3] Compared to one year ago, how would you rate your health in general now? [18]	×		×	×	×	×
	EQ-5D [20]	×		×	×	×	×
2.3 ADL/Physical functioning							
Disability in ADLs	Modified Katz Index Scale [16, 17]	×		×	×	×	×
Independency in walking	FAC [42]	×	×	×	×	×	×
Mobility	Could you walk outside for 5 minutes two weeks before admission/currently? And how often did/do you do physical activity two weeks before admission/currently? [19]	×		×	×	×	×
Falls	Have you fallen once or more in the past (six) month(s)? If yes, how many times? [25]	×		×	×	×	×
Pain	NRS pain [35]	×	×	×	×	×	×
Fatigue	NRS fatigue [37]	×	×	×	×	×	×
Impact of fatigue	MFIS-5 [38]				×	×	×
Sleep quality	PSQI [39]	×		×	×	×	×
Sleep medication	PSQI [39]	×		×	×	×	×
Daytime sleepiness	Do you currently suffer from daytime sleepiness? If yes, does this affect your daily living?	×	×	×	×	×	×
Polynocturia	Do you currently suffer from polynocturia? If yes, does this affect your daily living?	×	×	×	×	×	×
Dizziness	Do you currently suffer from dizziness? If yes, does this affect your daily living?	×	×	×	×	×	×
Shortness of breath	Do you currently suffer from shortness of breath? If yes, does this affect your daily living?	×	×	×	××	×	×
Hearing impairment	Do you experience difficulties with hearing, despite the use of a hearing aid?	×			×		×
Vision impairment	Do you experience difficulties with your vision, despite the use of glasses?	×			×		×
Nutrition	SNAQ [25, 41]	×		×	×	×	×
Dependency	Do you smoke? Do you use alcohol [19]?	×			×		×
Polypharmacy	Do you use five or more different medications [19]?	×			×		×
2.4 Health care utilization							
Readmission	Have you been hospitalized in the last (six) month(s)? If yes, for how many days? [18]	×*			×	×	×
Nursing home admission	Have you had a nursing home admission in the last month? If yes, for how many weeks totally? [18]				×	×	×
Consult physiotherapist and/or occupational therapist	Have you had a consultation with your physiotherapist and/or occupational therapist in the last month? If yes, how many times?				×	×	×
Consult general practitioner	Have you had a consultation with your general practitioner in the last month? If yes, in the evening, night or weekend and how many times totally? [19]				×	×	×
Home care	Do you use home care? If yes, care assistance and/or domestic help and how many hours per week [19]				×	×	×
3. Physical performance tests							
Handgrip strength	Jamar® [59–61]	×	×	×	×		×
Mobility	DEMMI [45]	×	×	×	×		×
Agility	CSR [47]	×	×	×	×		×
Balance, strength, and gait	SPPB [46]	×	×	×	×		×
Walking distance	2MWT [49]	×	×	×	×		×
Body composition	BIA (Bodystat Quadscan 4000) [50]	×	×	×	×		×
Activity tracker	Fitbit Flex [51]	×	×	×	×		×
	Question or instrument	H_1_	H_2_/H_3_		P_1_	P_2_	P_3_
4. Blood parameters							
Inflammation markers	CRP [52]	×	×				
	WBC diff	×	×				
	TNF-α [53–55]	×	×				
	IL-6 [53–55]	×	×				
	IL-8 [55]	×	×				
Mortality	Date of death						×*

*Note:* H_1_ = within 48 h after admission; H_2_ = during hospitalization on Monday, Wednesday, and/or Friday; H_3_ = at discharge; P_1_ = one month post-discharge (home visit); P_2_ = two months post-discharge (by telephone); P_3_ = three months post-discharge (home visit);

×* = Data will be obtained from medical record;

*CCI* Charlson Comorbidity Index, *MEWS* Modified Early Warning Score, *MMSE* Mini Mental State Examination, *CAM* Confusion Assessment Method, *NRS* Numeric Rating Scale, *STAI-6* State Trait Anxiety Inventory-6, *GDS-15* Geriatric Depression Scale-15, *ALCOS-12 Algemene Competentie Schaal*-12 (General Self-Efficacy Scale), *EQ-5D* EuroQol-5D, *FAC* Functional Ambulation Categories, *MFIS-5* Modified Fatigue Impact Scale-5, *PSQI* Pittsburgh Sleep Quality Index, *SNAQ* Short Nutritional Assessment, *DEMMI* De Morton Mobility Index, *CSR* Chair Sit and Reach test, *SPPB* Short Physical Performance Battery, *2MWT* 2 Minute Walking Test, *BIA* Bioelectrical Impedance Analysis, *CRP* C-Reactive Protein, *WBC diff* White Blood Cell Differential, *TNF-α* Tumor Necrosis Factor-α, *IL-6* Interleukin-6, *IL-8* Interleukin-8

1. **Medical and demographical data**
  *Socio-demographic characteristics.* Socio-demographic data include age, gender, date and time of admission, highest level of education, ethnicity, marital status and living arrangement.
  *Geriatric conditions.* A comprehensive geriatric assessment (CGA) will be collected, which will provide insight in the pre-illness determinants such as polypharmacy, substance use, incontinence, and vision- and hearing impairments.
  *Chronic conditions.* The number and severity of comorbidities will be scored with the Charlson Comorbidity Index [21]. Depending on the risk of mortality, each condition is assigned a score of 1, 2, 3, or 6. Higher scores indicate a greater risk of mortality.
  *Severity of acute illness.* The severity of the acute illness will be measured with the Modified Early Warning Score (MEWS). The MEWS is based on 1] respiratory rate; 2] heart rate; 3] systolic and diastolic blood pressure; 4] level of consciousness; 5] temperature, and; 6] oxygen saturation [22].
  **Personal interviews/self-report data**
  **(2.1) Cognitive functioning**
  *Cognitive impairments.* The most commonly used Mini Mental State Examination (MMSE) will be applied to classify the severity of a cognitive impairment. It is a validated 23-item screening of cognitive impairment. The MMSE consists of a series of questions and tests, which assess different mental abilities, including memory, attention, language, and planning. Cognitive impairment is defined as a score of 23 or less on the MMSE [23].
  *Delirium.* The Confusion Assessment Method (CAM) will be used to identify the presence of delirium. The CAM consists of four features: 1] acute onset and fluctuating course; 2] inattention; 3] disorganized thinking, and 4] altered level of consciousness. The diagnosis of delirium requires the presence of both features 1 and 2, and the presence of either feature 3 or 4 [24]. Furthermore, we want to assess the risk for developing delirium with the following statements of the Dutch Safety Management Programme (*Veiligheidsmanagementsysteem* (VMS)): 1] the patient needs help with self-care, 2] the patient has previously undergone a delirium, and 3] the patient has a cognitive impairment such as dementia [25, 26].
  **(2.2) Behavioral and psychosocial functioning**
  *Fear of falling.* A Numeric Rating Scale (NRS) will be applied to measure fear of falling, in which a participant selects a whole number (0–10 integers). Zero represents no fear of falling and ten the worst possible fear of falling.
  *Depression.* The Geriatric Depression Scale-15 (GDS-15) will be used to measure symptoms of depression (Cronbach’s α = 0.75 [27]). The GDS-15 is a self-report scale of 15 items on a binary (yes/no) scale and assesses symptoms over the preceding week. The total score is the sum of the 15 items (range 0–15 points, higher scores indicating more depression). The following categories of the GDS-15 will be used: a score of 0 to 4 will be considered ‘normal’, a score of 5 to 8 a ‘mild depression’, 9 to 11 a ‘moderate depression’, and 12 to 15 a ‘severe depression’ [28].
  *Apathy.* Three items of the GDS-15 will be used to measure apathy (sensitivity of 69 % and specificity of 85 % [29]). The three apathy items include the following questions: 1] “Do you prefer to stay at home, rather than going out and doing new things?”; 2] “Have you dropped many of your activities and interests?” and; 3] “Do you feel full of energy? Higher scores indicate more apathy. A score of ≥2 points is indicative for apathy [29].
  *Anxiety.* The State-Trait Anxiety Inventory-6 (STAI-6) will be used to measure anxiety symptoms (Cronbach’s α = 0.79-0.81 [30]). The STAI-6 is a short-form of the 20-item state scale of the Spielberger State-Trait Anxiety Inventory (STAI) [31], that maintains results that are comparable with this full-form [30]. It consists of six items on a 4-point Likert scale (1] not at all/almost never; 2] somewhat/sometimes; 3] moderately so/often, and; 4] very much so/almost always). Furthermore, it remains sensitive to different levels of anxiety.
  *Perceived self-efficacy.* The General Self Efficacy Scale (In Dutch: *Algemene Competentie Schaal* (ALCOS-12)) will be used to measure general perceived self-efficacy (Cronbach’s α = 0.78 [32]). It is based on the Self-Efficacy Scale [33] and is a Dutch translated self-report rating scale of 12 items on a 5 point Likert scale (1] strongly disagree; 2] disagree; 3] no disagreement/agreement; 4] agree and; 5] strongly agree). The ALCOS-12 includes three subscales: competence (Cronbach’s α = 0.72), perseverance in adversity (Cronbach’s α = 0.67), and taking initiative (Cronbach’s α = 0.74) [32]. The total score is the sum of the 12 items (range 12–60), whereby the following categories of the ALCOS-12 will be used: a score of 12 to 38 will be defined as a ‘low competence level’, a score of 39 to 54 as ‘average’ and 55 to 60 as ‘high’ [34].
  *Health-Related Quality of life.* The EuroQol-5D (EQ-5D), a widely used preference based generic health-related quality of life (HRQoL) instrument with well-established psychometric properties will be administered [20]. The EQ-5D consists of five dimensions: 1] mobility; 2] self-care; 3] usual activities; 4] pain/discomfort and; 5] anxiety/depression. These dimensions have three response choices (no problems; some problems or; severe problems). Moreover, the following questions will be used to measure quality of life: 1] “In general, how is your quality of life (participants answer the item with one of five possible responses: excellent; very good; good; moderate or; bad)?”; 2] “How would you grade your life at this moment, with a range between 0 and 10?” and; 3] “Compared to one year ago, how would you rate your health in general now (five response choices: much better; slightly better; much the same; slightly worse or; much worse)?” [18].
  **(2.3) Physical functioning**
  *Dizziness, polynocturia and shortness of breath.* Symptoms of dizziness and shortness of breath will be assessed by asking: “Do you suffer from polynocturia/dizziness/shortness of breath at this moment? If yes, does this affect your daily functioning?”
  *Pain.* A gold standard of pain intensity measurements, the Numeric Rating Scale (NRS), will be applied to measure pain. The NRS for pain is a validated continuous scale with a score range between 0 and 10 (0 represents no pain and 10 the worst possible pain) [35, 36].
  *Fatigue.* The Numeric Rating Scale (NRS), will be used to measure fatigue. The NRS for fatigue is a continuous scale with a score range between zero and ten (zero represents no pain and ten the worst possible fatigue) [37].
  *Impact of fatigue.* The abbreviated version of the 21-item Modified Fatigue Impact Scale (MFIS) will be used to quantify the impact of fatigue. The short version consists of five items that are divided into three subscales: physical- (2 items), cognitive- (2 items), and psychosocial functioning (1 item) subscale. An example of a MFIS-5 statement is: “Because of my fatigue during the past four week, I have been less alert.” The total score of the MFIS-5 is the sum of the raw scores on a 5-point Likert scale (0] never; 1] rarely; 2] sometimes; 3] often, and; 4] almost always). Higher scores indicate greater fatigue [38].
  *Sleep.* The Pittsburgh Sleep Quality Index (PSQI) will be utilized to measure two components of sleep: sleep quality and sleep medication. Sleep quality will be quantified by asking: “During the past month, how would you rate your sleep quality overall?” Sleep medication will be measured by asking: “During the past month, how often have you taken medicine (prescribed or “over the counter”) to help you sleep?” The score of sleep quality and sleep medication have a range of 0 (better) to 3 (worse) [39]. In addition, we measure daily sleepiness on a binary scale (yes/no) with the following question: “Do you currently suffer from daytime sleepiness? If yes, does this affect your daily living?”
  *Nutrition.* The widely used Short Nutritional Assessment Questionnaire (SNAQ) will be applied to identify malnourished hospital patients (Cronbach’s alpha = 0.58 [40]) [25, 26]. The total score of the SNAQ is the sum of the raw scores, whereby the following categories of the SNAQ will be used: a score of 0 to 1 will be defined as ‘no malnutrition’, a score of 2 as ‘moderate malnutrition’ and a score of 3 as ‘severe malnutrition’ [41].
  *ADL functioning.* The 15 items modified Katz-ADL index will be used to measure physical functioning [16, 17]. The modified Katz-ADL index consists of statements of their independency in performing basic Activities of Daily Living (ADL) and Instrumental Activities of Daily Living (IADL) (formulated in two versions on a binary (yes/no) scale: two weeks before admission or currently).
  *Mobility.* The Functional Ambulation Categories (FAC) will be used to classify mobility, using six categories: a category of 1 will be defined as ‘independent unlimited’, a category of 2 as ‘independent limited’ and categories 3 to 5 as ‘dependent’. Allocation to these last categories is based on levels of assistance and supervision needed [42]. Furthermore, we will measure mobility with two questions in according to the Comprehensive Geriatric Assessment (CGA) of the Dutch Society of Clinical Geriatrics (NVKG, 2012): 1] “Were you able to walk outside the house for five minutes (formulated in two versions: two weeks before admission or currently)?”, and; 2] “How often did/do you perform physical activity two weeks before admission/currently [19]?”
  *Falls.* To measure the number of falls in the past (six) month(s) the following question of the VMS will be used: “Have you fallen once or more in the past (six) month(s)? If yes, how many times [25, 26]?”
  **(2.4) Health care utilization**
  *(Re)admission(s).* Any (re)admission(s) to the hospital will be measured. We will search the medical record for (re)admission(s) in the same hospital six months before hospitalization and during three months post-discharge, and we will also retrieve this information by self-report at P_1_-P_3_ with the following self-report question: “Have you been hospitalized in the last month? If yes, for how many days [18]?” Data that will be collected out of the hospital system are: date of admission and discharge for any readmission, whether the admission was planned or unplanned and the reason for the readmission.
  *Nursing home admission(s).* The amount of nursing home admission or whether they were admitted to the nursing home and the length of stay will be measured with the subsequent question: “Have you had a nursing home admission in the last month? If yes, for how many weeks totally [18]?”
  *Consult of physical therapist and/or occupational therapist.* The amount of consults of a physiotherapist and/or occupational therapist will be measured by asking: “Have you had a consultation with your physical therapist and/or occupational therapist in the last month? If yes, how many times?”
  *Consult general practitioner.* The amount of consults of a general practitioner will be measured by asking: “Have you had a consultation with your general practitioner in the last month? If yes, in the evening, night or weekend and how many times in total [19]?”
  *Home care.* The use of home care will be measured with the subsequent question: “Do you use home care? If yes, care assistance and/or domestic help and how many hours per week [19]?” A distinction will be made between household help from a nursing aid, and help from a registered nurse.
2. **Physical performance tests**
  *Handgrip strength.* The hand grip strength will be measured with the widely used Jamar® grip strength dynamometer (Lafayette Instrument Company, USA). The handgrip strength test is used to provide an objective index of general upper body strength. Handgrip strength is a reliable instrument (good to excellent test-retest reproducibility and excellent inter-rater reliability) to indicate skeletal muscle mass [43]. Participants will perform the task thrice with each hand. The highest score from either hand will be used and registered in the eCRF. Normative values of adults are described in a study of Mathiowetz [44].
  *Mobility.* To measure the mobility we will use the 15-item Morton Mobility Index (DEMMI). Subjects will be asked to perform several mobility tasks, in the order of bed, chair, stand, and walking activities to maximize patient safety, which will result in an ordinal raw score (range: 0–19). The ordinal raw score will be converted into a total interval DEMMI score (range: 0 to 100 points). Moreover, the DEMMI has a hierarchical structure, and thus each assessed participant can be evaluated. Higher scores indicate a better mobility performance [45].
  *Balance, strength, and gait speed.* The Short Physical Performance Battery (SPPB) will be applied to measure the balance, strength and gait speed. Participants will be asked to stand with their feet in various balance positions, walk a distance of four meter and to rise from a chair and return to the seated position five times as quickly as possible. Higher scores indicate a better performance [46].
  *Back and hamstring flexibility.* The Chair Sit and Reach (CSR) test will be used as a measure of flexibility. Participant will be asked to extend one leg as straight as possible, hands on top of each other, and then to reach to his/her foot as far as possible. Lower distances between the tip of his/her toes and their extended fingers indicating a higher back and hamstring flexibility [47, 48].
  *Walking distance.* The 2 Minute Walking West (2MWT) will be applied to measure the maximal walking distance in meters. Participants will be asked to walk back and forth along a premeasured corridor of 15 meter in two minutes. Longer walking distances indicating a better walking capacity [49].
  *Body composition.* The Bioelectrical Impedance Analysis (BIA) (Bodystat Quadscan 4000) will be used as method for estimating body composition, in particular fat-free mass (FFM) and high fat mass (FM). Electrodes will be attached to the ankle and wrist. A small electric signal will circulate, which measures the resistance and reactance of this electrical signal in the human body [50].
  *Activity level.* The Fitbit Flex will be applied to monitor the sleep quality, measure motion patterns, determine the calories burned, distance traveled, and steps taken [51]. Participants will be asked to wear the Fitbit Flex from hospital admission up to one and a half weeks post-discharge.
3. **Blood parameters**
  *Inflammation markers.* Inflammation markers, such as C-Reactive Protein (CRP) [52], Tumor Necrosis Factor-α (TNF-α), the interleukins IL-6 [53–55] and IL-8) [55], and White Blood Cell Differential (WBC diff), will be determined from blood plasma and serum. Blood will be collected during the customary laboratory rounds during hospitalization. Venous blood will be collected in 4.5 ml EDTA and serum vacutainers. Samples will be centrifuged and stored at −80 C° until analysis. Sample handling and analyses will be performed according to ISO standards.

### Planned statistical analyses

Data will be analyzed in accordance with the research questions outlined in the introduction, applying appropriate General Linear Models (e.g., linear regression, repeated measures ANOVA/ANCOVA) as well as log-linear models (e.g., logistic regression in case of binary outcomes). Mortality, a (censored) numerical outcome, will be tested using survival analysis. The global α level will be set at 0.05 with hypothesis-wise adjustment for multiple testing. All analyses will be performed using SPSS version 22.0 [56]. Castor Electronic Data Capture (EDC) will be used to build electronic Case Report Forms (eCRFs) for save and valid data collection.

Primary endpoint in the study will be HAD as measured with the Katz-ADL index score. For multivariable analyses (General Linear models and log-linear models) a custom 10:1 case-to-outcome ratio is utilized as a maximum. Utilizing a repeated measures design, power calculations imputing a conservative α level of 0.01 yielded a power of 95 % for associations of a small effect-size (Cohen’s f = 0.069), whereas a power of 80 % was established for associations with an effect-size of 0.058 (Cohen’s f).

## Discussion

More than 30 % of the older patients experience hospitalization-associated disability (HAD) after acute hospitalization [1, 3, 4], which implies the loss of ability to perform one or more of the basic ADLs [6]. HAD is the leading cause of functional decline at older age [4]. With a higher number of older persons and an increasing life expectancy, there is an urgent need to unravel the potential mechanisms behind HAD as well as how the mechanisms can be influenced. To our knowledge, the Hospital-ADL study is the first study that investigates cognitive, behavioral, psychosocial, physical, and biological factors simultaneously. The current study will provide novel information regarding possible underlying mechanisms behind HAD within the critical period of three months post hospitalization, which is expected to lead to the development of interventions that can prevent or restore HAD.

### Ethics approval and consent to participate

The study is approved by the Institutional Review board of the Academic Medical Center (AMC) in The Netherlands (Protocol ID: AMC2015_150). Written informed consent is obtained from all participants before inclusion. The research is performed according to the Dutch Medical Research Involving Human Subjects Act and principles of the Declaration of Helsinki (1964).

## Abbreviations

2MWT: 2 min walking test
ADL: activities of daily living
ALCOS-12: *Algemene Competentie Schaal-*12 (Perceived Self-Efficacy)
BIA: bioelectrical impedance analysis
CAM: confusion assessment method
CCI: Charlson comorbidity index
CGA: comprehensive geriatric assessment
CRF: case report form
CRP: C-Reactive Protein
CSR: Chair Sit an Reach test
DEMMI: De Morton Mobility Index
EQ-5D: EuroQoL-5D
FAC: functional ambulation categories
GDS-15: geriatric depression scale-15
HAD: hospitalization-associated disability
Hospital-ADL study: Hospital-Associated Disability and impact on daily Life study
IADL: instrumental activities of daily living
IL- 8: interleukin- 8
IL-6: interleukin- 6
MDS: minimal dataset
MEWS: modified early warning score
MFIS-5: modified fatigue impact scale-5
MMSE: mini mental state examination
NRS: numeric rating scale
PSQI: Pittsburgh Sleep Quality Index
SNAQ: short nutritional assessment
SPPB: short physical performance battery
STAI-6: state trait anxiety inventory-6
TCB: transitional care bridge
TNF-α: tumor necrosis factor-α
VMS: safety management programme (In Dutch: *Veiligheidsmanagementsysteem*)
WBC diff: white blood cell differential
